# Identification of IGF2BP3 as an Adverse Prognostic Biomarker of Gliomas

**DOI:** 10.3389/fgene.2021.743738

**Published:** 2021-10-13

**Authors:** Chao Sun, Xin Zheng, Yingxin Sun, Ju Yu, Minfeng Sheng, Suji Yan, Qing Zhu, Qing Lan

**Affiliations:** Department of Neurosurgery, The Second Affiliated Hospital of Soochow University, Suzhou, China

**Keywords:** glioma bioinformatics analysis, M6A RNA methylation, post-transcriptional modification, prognosis, IGF2BP3

## Abstract

N6-methyladenosine (m^6^A) RNA modification can alter gene expression and function by regulating RNA splicing, stability, translocation, and translation. It is involved in various types of cancer. However, its role in gliomas is not well known. This study aimed to determine the prognostic value of the m^6^A RNA methylation regulator in gliomas and investigate the underlying mechanisms of the aberrant expression of m^6^A-related genes.mRNA expression profiles and clinical information of 448 glioma samples were obtained from The Cancer Genome Atlas and cBioportal. The expression of m^6^A-related genes in normal controls and low-grade glioma and glioblastoma was obtained from Gene Expression Profiling Interactive Analysis. Further, m^6^A-related gene expression and its relationship with prognosis were obtained through The Chinese Glioma Genome Atlas (CGGA). Multivariate Cox regression analyses were performed, and a nomogram was built with potential risk factors based on a multivariate Cox analysis to predict survival probability. Online tools such as Gene Set Enrichment Analysis, STRING, Cytoscape, and Molecular Complex Detection were applied for bioinformatics analysis and to investigate the underlying mechanisms of the aberrant expression of m^6^A-related genes. The multivariate Cox regression analysis found that higher expression levels of YTHDC2 and insulin-like growth factor 2 mRNA-binding protein 3 (IGF2BP3, also called IMP3) were independent negative and positive prognostic factors for overall survival (OS), respectively. Data from the CGGA database showed that IGF2BP3 expression increased when the tumor grade increased. Receiver operating characteristic (ROC) curve was used to evaluate the predictive specificity and sensitivity. The area under the ROC curve indicated that the OS prediction was 0.92 (1-year) and 0.917 (3-years), indicating that m^6^A-related genes could predict patient survival. In addition, IGF2BP3 was closely related to the shorter survival period of patients. Copy number variation and DNA methylation, but not somatic mutations, might contribute to the abnormal upregulation of IGF2BP3 in gliomas. Significantly altered genes were identified, and the protein–protein interaction network was constructed. Based on the data presented, our study identified several m^6^A-related genes, especially IGF2BP3, that could be potential prognostic biomarkers of gliomas. The study unveiled the potential regulatory mechanism of IGF2BP3 in gliomas.

## Introduction

Gliomas are among the most common primary brain tumors in adults and account for more than 70% of malignant brain tumors (Gusyatiner and Hegi, 2018). Among gliomas, glioblastoma (GBM) is most aggressive and is characterized by its high proliferative potential, infiltrative growth, and high recurrence rate (Rajesh et al., 2017). The prognosis of patients with GBM remains poor; the progression-free survival of patients with GBM is only 6 months, with a median survival of 12–18 months (Ostrom et al., 2015). In recent years, the molecular targeted therapy has become a research hotspot in GBM therapy. Since GBM is highly heterogeneous, the effect of current therapies is limited, and no remarkable progress has been achieved yet. Therefore, precise therapeutic targets for treating gliomas are urgently needed. However, the natural characteristics of GBM remain far from fully understood. The molecular characteristics of GBM should be accurately determined to know how GBM initiates and develops (Dong and Cui, 2020).

Epigenetic abnormalities are considered to be one of the most essential carcinogenic mechanisms. At present, the epigenetic regulations of DNA and protein have been extensively studied, but RNA modification is still largely unknown. In recent years, more than 100 RNA modifications have been reported, including modifications within mRNAs, among which N6-methyladenosine (m^6^A) modification is the most prevalent internal modification in eukaryotic mRNAs (Wei et al., 1975; Wang et al., 2015). M^6^A modification is an invertible and dynamical RNA epitranscriptomic process regulated by m^6^A regulators, including writers, readers, and erasers (Zaccara et al., 2019).

The physiological significance of m^6^A modification of mRNA has only been appreciated in recent years. Moreover, the role of m^6^A in cancer is starting to be revealed (He et al., 2018). Accumulating evidence reveals the fact that m^6^A modification is essential for tumor initiation, development, metastasis, and stem cell characteristics in various types of cancer. However, the cellular and functional dynamics of m^6^A RNA modifications in the regulation of GBM initiation and progression remain largely unexplored. Identifying writers, readers, and erasers of m^6^A modification and developing the m^6^A-sequencing (m^6^A-seq) technology set the foundation for exploring the roles of m^6^A mRNA modification in cancer biology (Cui et al., 2017).

In this study, the mRNA expression profiles and the clinicopathological features of 448 glioma samples were obtained from The Cancer Genome Atlas (TCGA) and cBioportal. We analyzed the expression of these m^6^A-related genes in gliomas with different clinicopathological features of their TCGA datasets. Insulin-like growth factor 2 mRNA-binding protein (IGF2BP) 3 was selected based on survival and prognosis analysis. Multivariate Cox regression analyses were performed, and a nomogram was built with potential risk factors based on a multivariate Cox analysis to predict survival probability. Based on the data presented, we proposed a vital role and potential regulatory mechanism of insulin-like growth factor 2 mRNA-binding protein 3 (IGF2BP3, also called IMP3) in gliomas.

## Materials and Methods

### Expression and Genetic Alterations of m^6^A-Related Genes in Gliomas

The mRNA expression profiles and the clinical information related to 335 cases of low-grade glioma (LGG) and 113 cases of glioblastoma (GBM) were obtained from UCSC Xena (http://xena.ucsc.edu/) and cBioportal (http://www.cbioportal.org). The expression of m^6^A-related genes in normal controls and LGG and GBM was obtained from Gene Expression Profiling Interactive Analysis (GEPIA). The genetic changes and copy number variation (CNV) data of m^6^A-related proteins were obtained through cBioportal. IGF2BP3 gene expression and its relationship with prognosis were obtained through The Chinese Glioma Genome Atlas (CGGA) (http://www.cgga.org.cn).

### Survival Analysis and Immune Score

The cutpoint value of each gene was calculated using the “survival” and “survminer” R packages. (https://CRAN.R-project.org/package=survminer). In brief, the “survminer” package was used to determine the optimal cutoff value for assorting high expression and low expression. The cutpoint of the immune score was obtained using the X-tile 3.6.1 software (Yale University School of Medicine, CT, United States) by the aforementioned method.

### Analysis of IGF2BP3 in the CGGA database

To further explore the role of IGF2BP3 in gliomas, the CGGA data portal (http://www.cgga.org.cn) was used to analyze the expression of IGF2BP3 in glioma samples, and the survival of patients with the high expression level of IGF2BP3.

### Univariate and Multivariate Analyses

Multivariate Cox regression analysis was used to screen for regulators significantly related to the overall survival (OS) probability of glioma samples. The rms R software package was used to perform nomogram analysis by incorporating factors significantly related to the OS of patients with glioma into multivariate analysis. The time-dependent receiver operating characteristic (ROC) curve was used to evaluate the predictive value of the prognostic gene signature for OS using the R package “timeROC,” and the specificity and sensitivity of the ROC curve were calculated by “timeROC” [pmid 10877287].

### Mutation, CNV, and Methylation Analysis of IGF2BP3

IGF2BP3 mRNA expression, somatic mutation, CNV, and DNA methylation were obtained by exploring the UCSC Xena browser (https://xenabrowser.net/heatmap/) and cBioportal for cancer genomics.

### Differential Gene Expression Analysis

Patients with glioma were divided into low-IGF2BP3 group and high-IGF2BP3 group according to the cutoff value in survival analysis. Gene differential expression analysis was performed using the Limma R package. Further, log2-fold change (FC) > 2 and adjusted *p* value < 0.05 were used to obtain differentially expressed mRNAs. Gene Set Enrichment Analysis (GSEA) was used to inspect the signaling pathways involved in the high or low expression of IGF2BP3. Protein–protein interaction (PPI) networks were constructed using the STRING database, and Cytoscape software was used for visualization. The Molecular Complex Detection (MCODE) plugin of the Cytoscape software was used for cluster analysis on the functional modules in the protein interaction network. Cytoscape plugin cytoHubba was used to identify hub genes.

### Statistical Analysis

Statistical analysis was performed using R software V3.5.1 and GraphPad Prism v7.0. Continuous variables were described as mean ± standard deviation. Pearson *χ* ^2^ test, Fisher’s exact test, Mann–Whitney test, or Pearson correlation test was used as appropriate. Kaplan–Meier curves and log-rank test were used for survival difference analysis. Multivariate Cox regression analysis was used to analyze the genes related to prognosis. The adjusted *p* value was used to identify differentially expressed genes, while the *p* value was used for the other analysis. A *p* value < 0.05 indicated a statistically significant difference.

## Results

### Expression and Genetic Alterations of m^6^A-Related Genes

**FIGURE 1:**
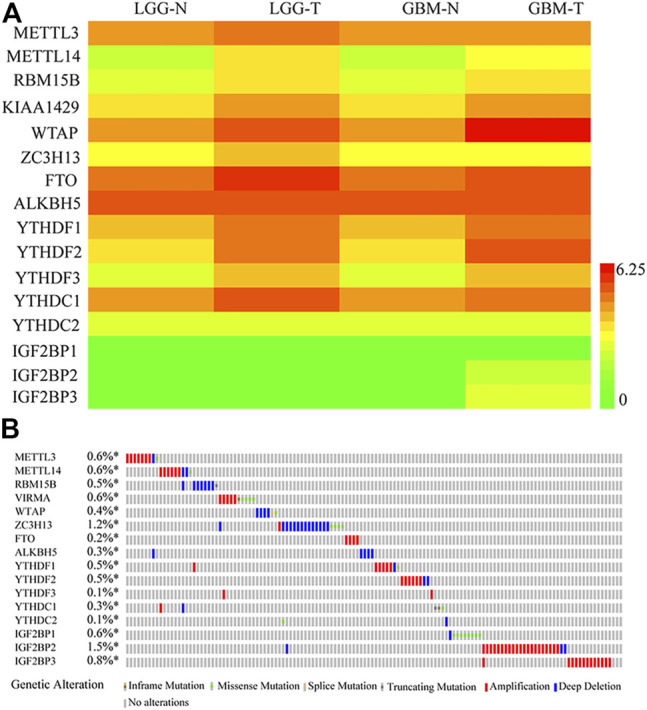
Expression and genetic alterations in the m^6^A-related genes in gliomas. **(A)** Expression profiles of the m^6^A-related genes in the TCGA-LGG and TCGA-GBM datasets. **(B)** Genetic alteration profiles of the m^6^A-related genes. The data were from the cBioportal for Cancer Genomics. N, Normal; T, tumor.

Given the critical role of m^6^A regulators in tumorigenesis and progression, we comprehensively analyzed the relationship between 16 m^6^A methylation regulators in glioma and normal samples using the TCGA dataset. The mRNA expression profiles and the clinical information related to 335 cases of LGG and 113 cases of glioblastoma (GBM) were obtained from TCGA. The expression levels of 16 m^6^A regulatory genes in glioma and para-tumor normal tissues are shown with a heatmap. As shown in Figure 1A, most m^6^A RNA methylation regulators had different expression levels in gliomas compared with para-tumor normal tissues. To investigate the role of m^6^A RNA methylation regulators in patients with glioma, we accessed the profiles of m^6^A RNA methylation−related genetic alteration. Genetic alterations were gathered from the TCGA in cBioPortal. Some genetic alterations of m^6^A-related genes were found, as shown in Figure 1B; amplification and deep deletion mutations were most common; and ZC3H13, IGF2BP2, and IGF2BP3 were the most frequently altered m^6^A-related gene.

**FIGURE 2:**
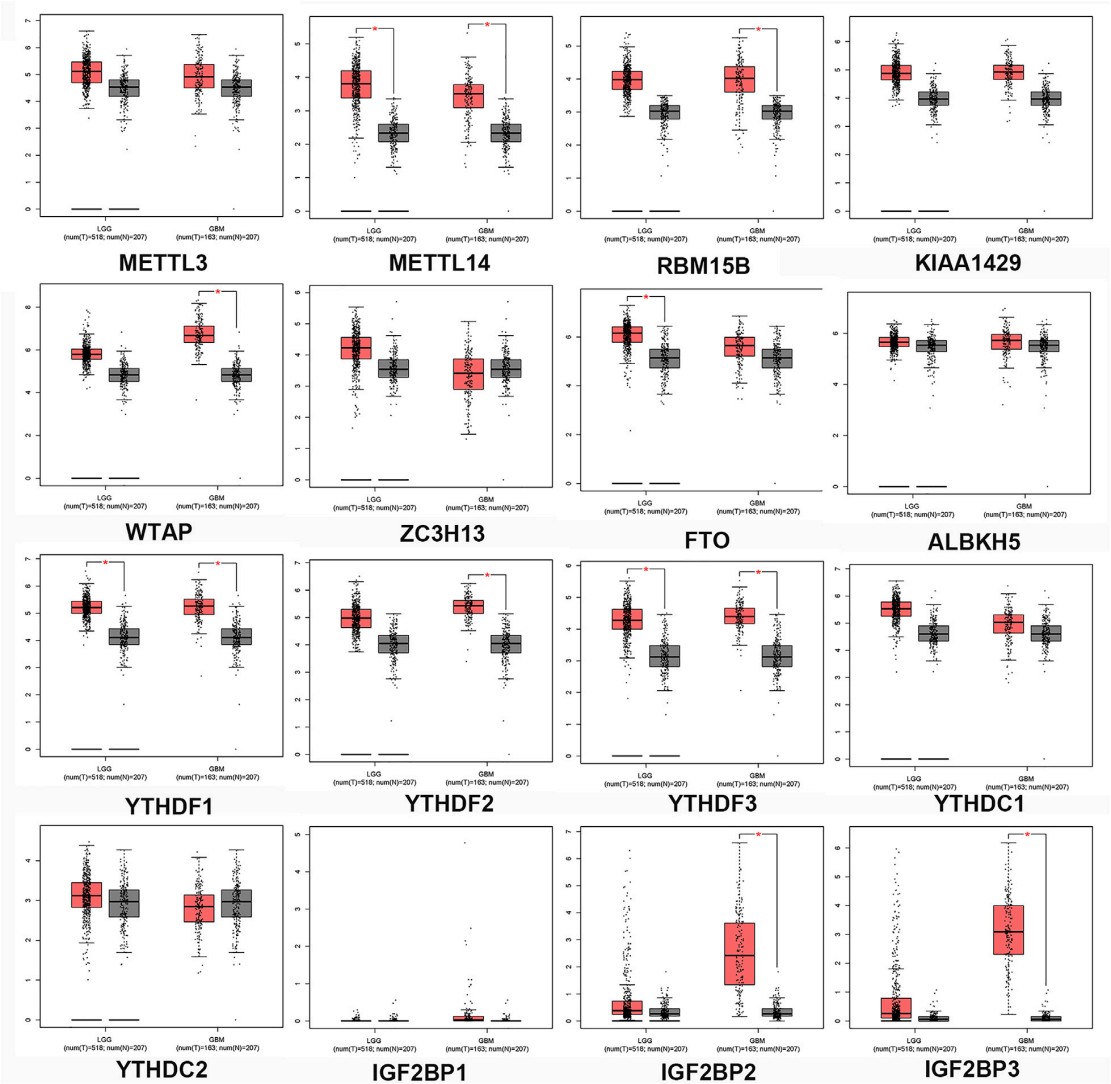
Expression profiles of the m^6^A-related genes in gliomas. The mRNA expression of m^6^A-related genes was analyzed using TCGA-LGG and TCGA-GBM datasets through GEIPA. T, Tumor tissues; N, normal tissues. *p* < 0.05, versus noncancerous brain tissues.

To better understand the interactions between m^6^A methylation regulators and gliomas, we also analyzed their correlations by GEPIA. GEPIA is a Web-based tool that provides fast and customizable functions based on TCGA and GTEx data. The expression of m^6^A-related genes in normal controls, LGG, and GBM was obtained from GEPIA and was presented in a boxplot (Figure 2). The expression levels of METTL14, FTO, YTHDF1, and YTHDF3 were significantly upregulated in LGG compared with normal tissues. GBM specimens displayed increased levels of RBM15B, WTAP, FTO, YTHDF2, YTHDF3, IGF2BP2, and IGF2BP3 when compared with normal brain controls. Importantly, the expression levels of IGF2BP3 were significantly upregulated.

### Relationship Between m^6^A-Related Genes and the Prognosis of Gliomas

**FIGURE 3:**
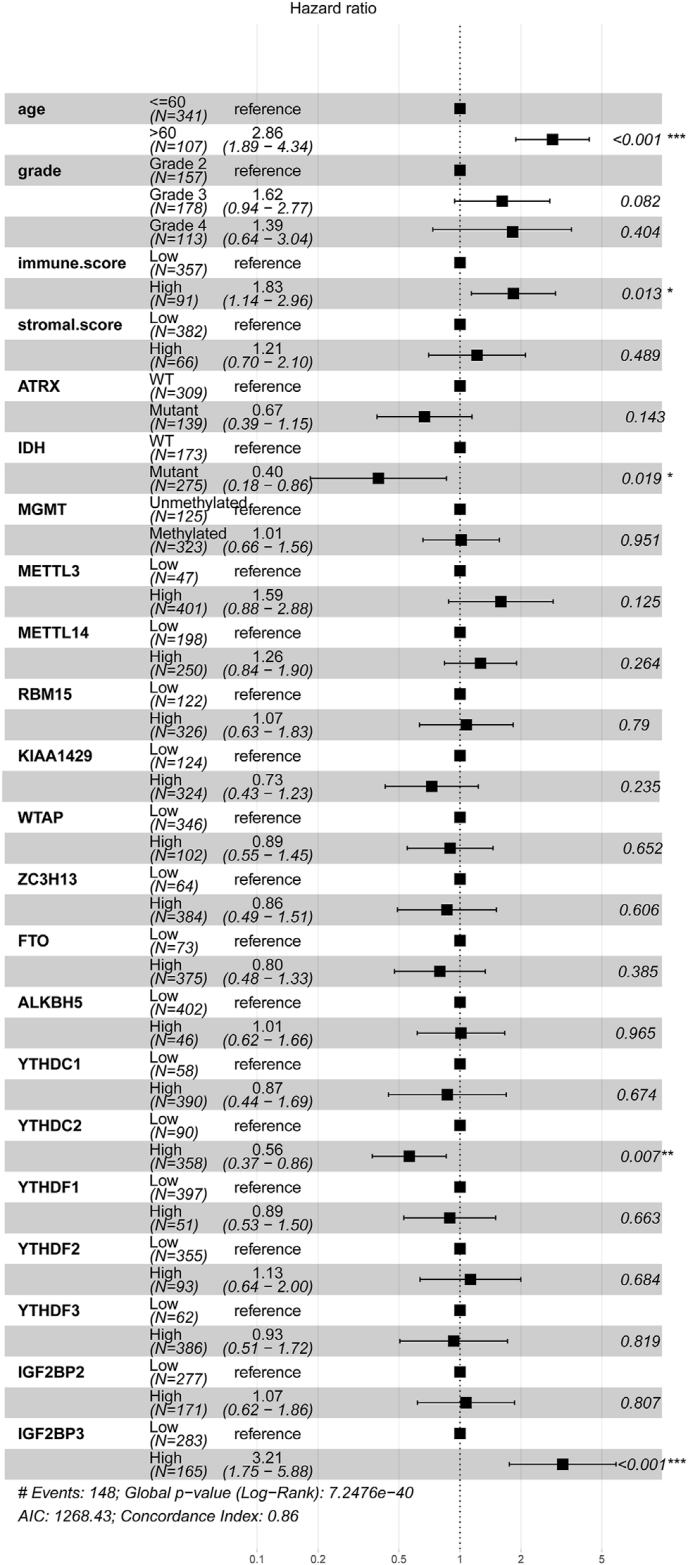
Association between m^6^A regulator and prognosis of glioma samples. The forest plot showed the result of multivariate Cox regression analysis for the association between m^6^A regulator’s expression, clinical features, and Kaplan–Meier estimated overall survival probability of glioma samples. Values within brackets represent the 95% confidence interval of the hazard ratio.

The basic clinical features of the 448 patients with glioma are summarized in Table 1. The results indicated that age, tumor grade, IDH mutation, ATRX mutation, MGMT methylation, and immune score were significantly associated with OS (*p* < 0.001). We then evaluated the association between the expression levels of m^6^A-related genes and the prognosis of gliomas. According to the best cutoff value of each gene, patients were divided into high-expression group and low-expression group. The univariate analysis suggested that except for METTL3, high expression of other m^6^A-related genes was associated with poor prognosis (Table 1). We then performed multivariate Cox regression analysis for these m^6^A-related genes and found that higher expression levels of YTHDC2 (*p* = 0.007) and IGF2BP3 (*p* < 0.001) were independent negative and positive prognostic factors for OS, respectively (Figure 3). Furthermore, we also validated the prognostic value of age (*p* < 0.001), immune score (*p* < 0.05), and IDH mutation (*p* < 0.05).

**TABLE 1:** Univariate analysis of overall survival using the Cox proportional-hazards regression model.

Variables	Number (overall = 448)	Univariate analysis		
		HR (95% CI)	C-index	*p*
Age (year)			0.704	
≤60	341			
>60	107	5.271 (3.721–7.467)		<0.001
Sex			0.497	
Female	196			
Male	252	1.085 (0.7814–1.507)		0.626
Grade			0.777	
Grade 2	157			
Grade 3	178	2.832 (1.726–4.647)		<0.001
Grade 4	113	16.881 (10.008–28.474)		<0.001
ATRX			0.647	
Wild type	309			
Mutant	139	0.3746 (0.2541–0.5522)		<0.001
IDH			0.783	
Wild type	173			
Mutant	275	0.125 (0.08737–0.1788)		<0.001
MGMT			0.64	
Unmethylated	125			
Methylated	323	0.3881 (0.2779–0.542)		<0.001
Stromal score			0.622	
Low	382			
High	66	4.258 (2.911–6.229)		<0.001
Immune score			0.659	
Low	357			
High	91	4.944 (3.446–7.095)		<0.001
METTL3			0.521	
Low	47			
High	401	0.6368 (0.3827–1.059)		0.0823
METTL14			0.6	
Low	198			
High	250	0.5728 (0.4137–0.7933)		0.008
RBM15			0.524	
Low	122			
High	326	1.624 (1.04–2.538)		0.033
KIAA1429			0.58	
Low	124			
High	324	0.5392 (0.3734–0.7787)		0.001
WTAP			0.66	
Low	346			
High	102	4.437 (3.133–6.286)		<0.001
ZC3H13			0.625	
Low	64			
High	384	0.2152 (0.1453–0.3187)		<0.001
FTO			0.644	
Low	73			
High	375	0.206 (0.1419–0.2991)		<0.001
ALKBH5			0.56	
Low	402			
High	46	2.65 (1.742–4.032)		<0.001
YTHDC1			0.631	
Low	58			
High	390	0.1485 (0.09767–0.2259)		<0.001
YTHDC2			0.573	
Low	90			
High	358	0.4984 (0.3493–0.7112)		0.0001
YTHDF1			0.565	
Low	397			
High	51	3.006 (1.986–4.549)		<0.001
YTHDF2			0.637	
Low	355			
High	93	3.387 (2.412–4.755)		<0.001
YTHDF3			0.59	
Low	62			
High	426	0.3448 (0.233–0.5102)		<0.001
IGF2BP2			0.722	
Low	277			
High	171	5.387 (3.816–7.605)		<0.001
IGF2BP3			0.733	
Low	283			
High	165	8.289 (5.709–12.03)		<0.001

**FIGURE 4:**
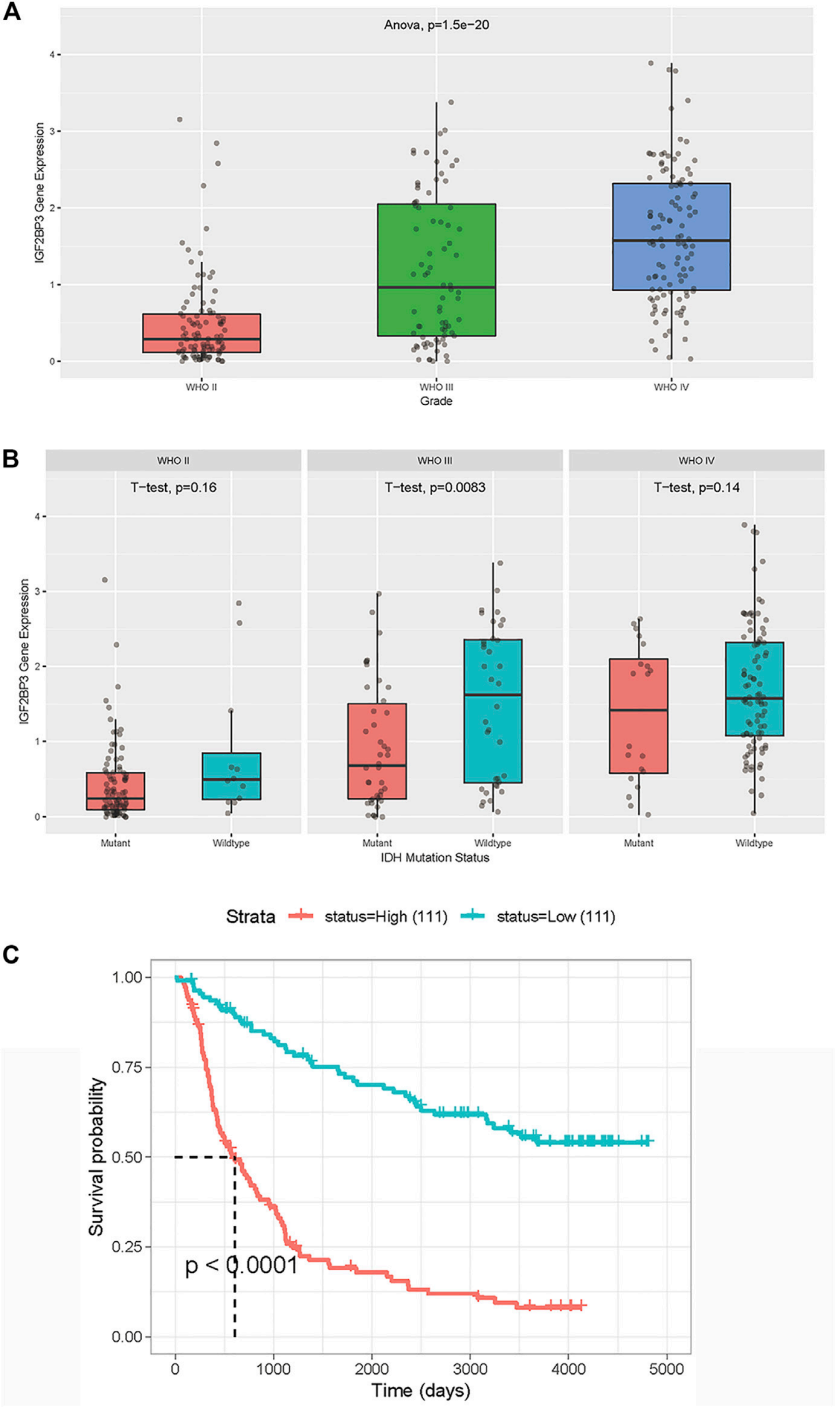
Validation of IGF2BP3 in Chinese patients with LGG and GBM derived from the CGGA dataset. **(A)** Association between IGF2BP3 expression and tumor grade. The *p* value was generated from analysis of variance). **(B)** Association between IGF2BP3 and IDH. The *p* value was generated from the *t* test. **(C)** Overall survival was analyzed in terms of high (red) or low (blue) expression of IGF2BP3 in the CGGA dataset. The *p* value was generated from the log-rank test.

To further study the expression of the most distinct IGF2BP3 gene in Chinese patients with glioma, the CGGA database was used. The IGF2BP3 expression was found to increase as the tumor grade increased (Figure 4A), indicating that IGF2BP3 might contribute to the progression of gliomas. We then analyzed the relationship between IDH status and expression levels of IGF2BP3 in different grades of gliomas (Figure 4B). A statistically significant difference was found between IDH-mutated and IDH-wide-type tumor tissues in Grade III gliomas, while no statistically significant difference was detected in Grades II and IV gliomas. Then we analyzed the OS of patients with glioma, and found that the OS of patients with high IGF2BP3 expression was significantly reduced (*p* < 0.001) (Figure 4C), which was consistent with the results of the TCGA.

### Association Between Risk Score and Glioma Epidemiological Statistics

**FIGURE 5:**
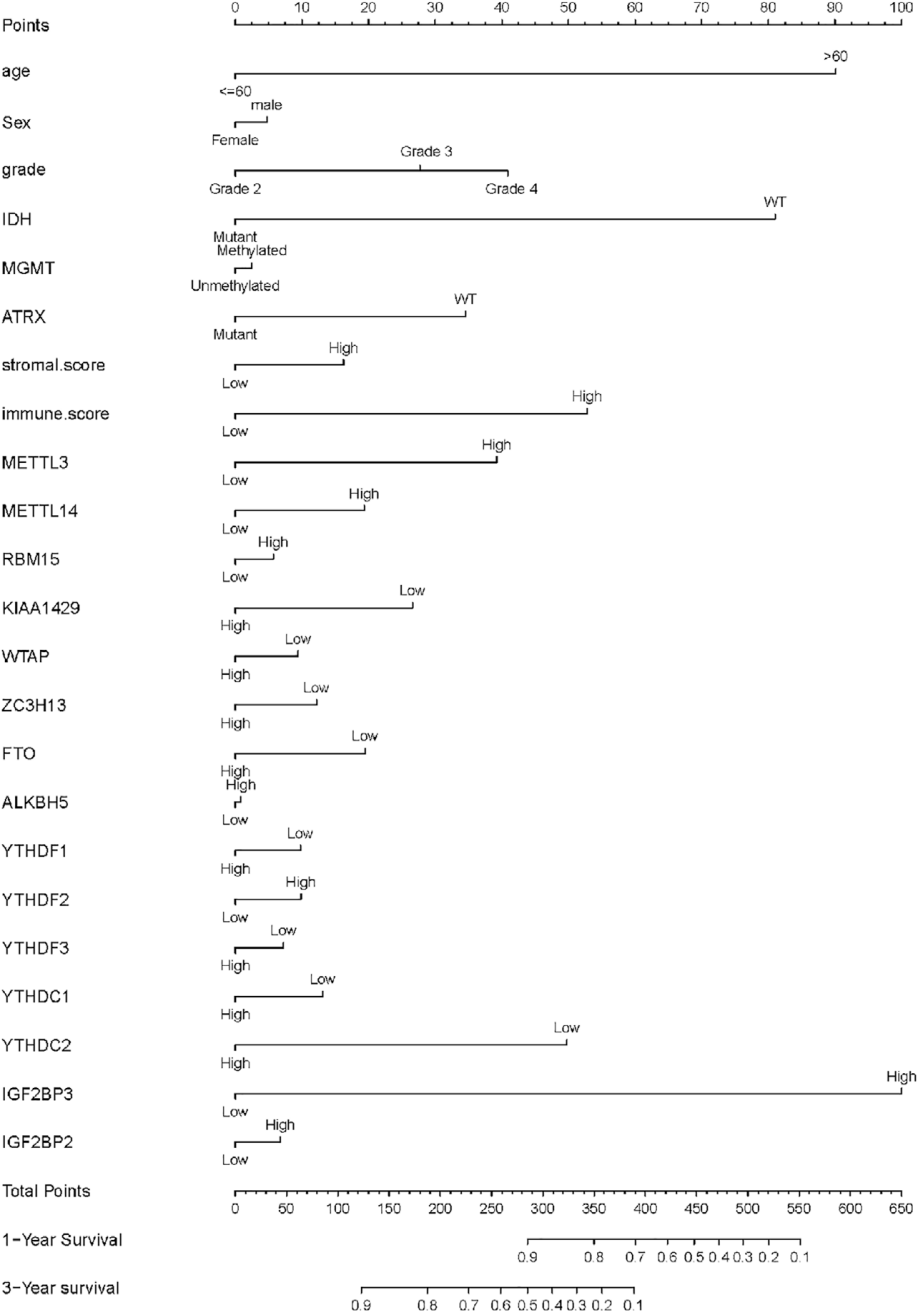
Glioma survival nomograms. For using the nomograms, the value for an individual patient is located on each variable axis, and a line is drawn upward to determine the number of points received for each variable value. The sum of these numbers is located on the Total Points axis, and a line is drawn downward to the survival axes to determine the likelihood of 1- or 3-years survival.

**FIGURE 6:**
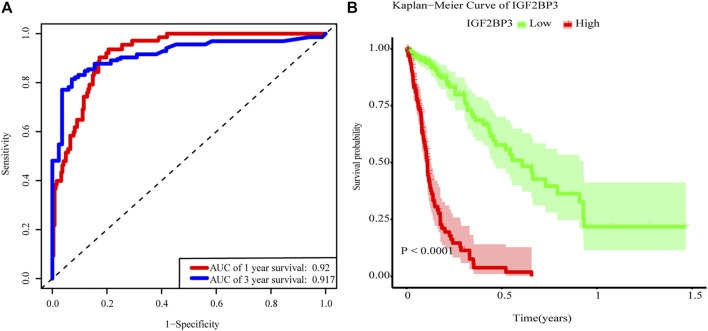
m^6^A-related gene model in prognosis. **(A)** ROC curves. AUC = 0.92 for 1-year (blue) and AUC = 0.917 for 3-years survival (red). **(B)** Overall survival was analyzed in high (red) or low (blue) expression of IGF2BP3 in the TCGA glioma dataset. The *p* value was generated from the log-rank test.

The main factors of multivariate Cox regression analysis, such as age, sex, grade, IDH, and risk score, were used to construct a nomogram to predict the probability of OS at 1 or 3 years, as shown in Figure 5. We could calculate the total points of each patient and then obtained the 1- year and 3-years survival rates. The higher the total points of a patient, the worse the prognosis. We further performed the ROC curve to evaluate the predictive specificity and sensitivity. The area under the ROC curve (AUC) indicated that the OS prediction was 0.92 (1-year) and 0.917 (3-years), indicating that IGF2BP3 could predict patient survival (Figure 6A). In addition, the curve of Kaplan–Meier revealed that the high expression of IGF2BP3 was closely related to the shorter survival period of patients (Figure 6B).

### Mutation, CNV, and Methylation Analysis of IGF2BP3

**FIGURE 7:**
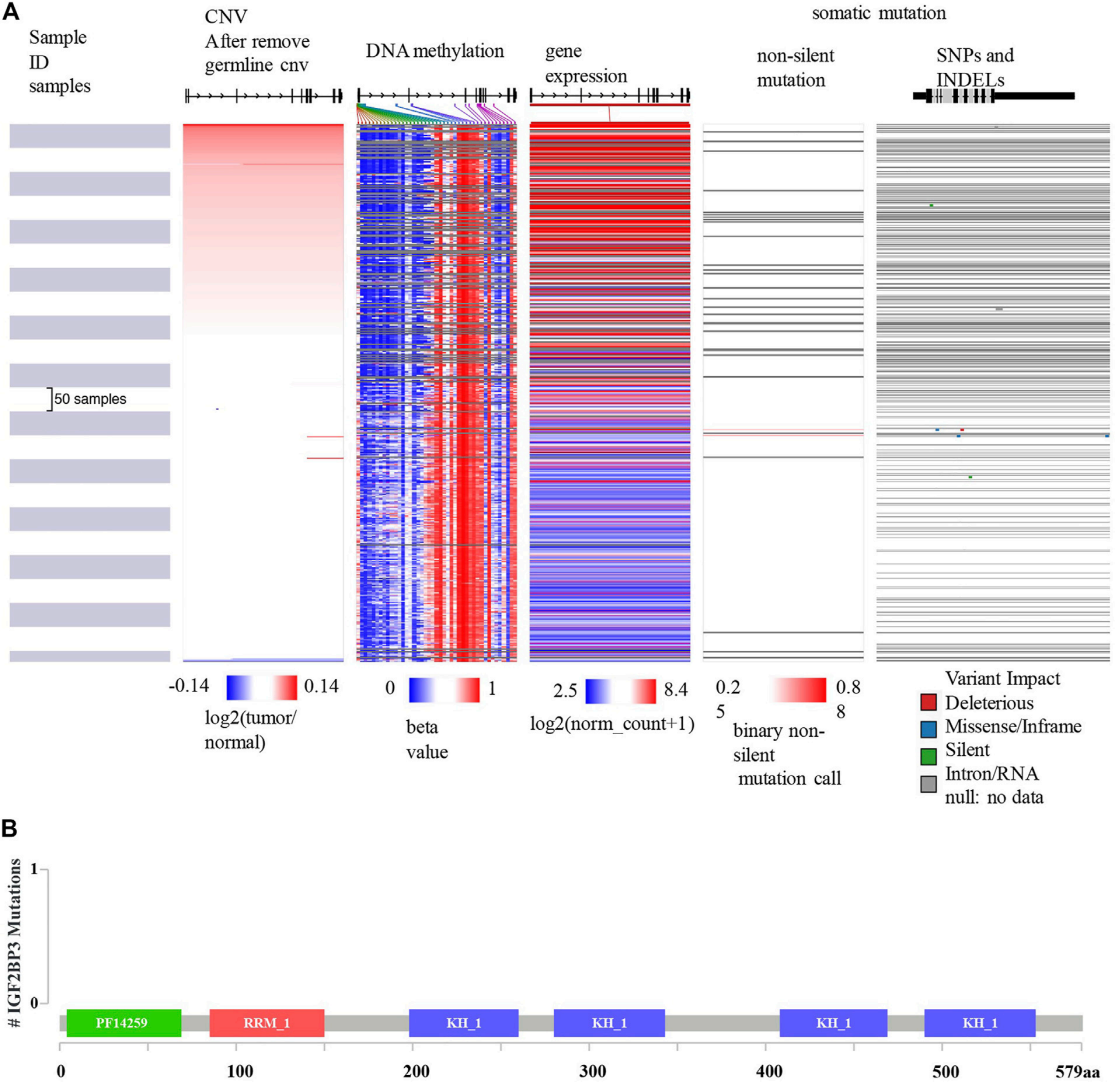
Mutation, CNV, and methylation analysis of IGF2BP3 in gliomas. **(A)** Heatmap showing the correlations between IGF2BP3 mRNA and somatic mutations, CNV, and methylation in gliomas via UCSCXena. **(B)** Correlation between IGF2BP3 mRNA and somatic mutation in gliomas via the cBioportal for Cancer Genomics.

Since IGF2BP3 is an independent prognostic factor of OS, we further analyzed the underlying mechanism of its dysregulation. As shown in Figure 7A, data from the UCSC Xena database showed that the expression of IGF2BP3 mRNA correlated with CNV and some DNA methylation sites, but not with somatic mutations. The mutation map from cBioportal for Cancer Genomics further confirmed that the dysregulation of IGF2BP3 in patients with glioma was not related to somatic mutations (Figure 7B).

### mRNA Closely Related to IGF2BP3 Expression in Gliomas

**FIGURE 8:**
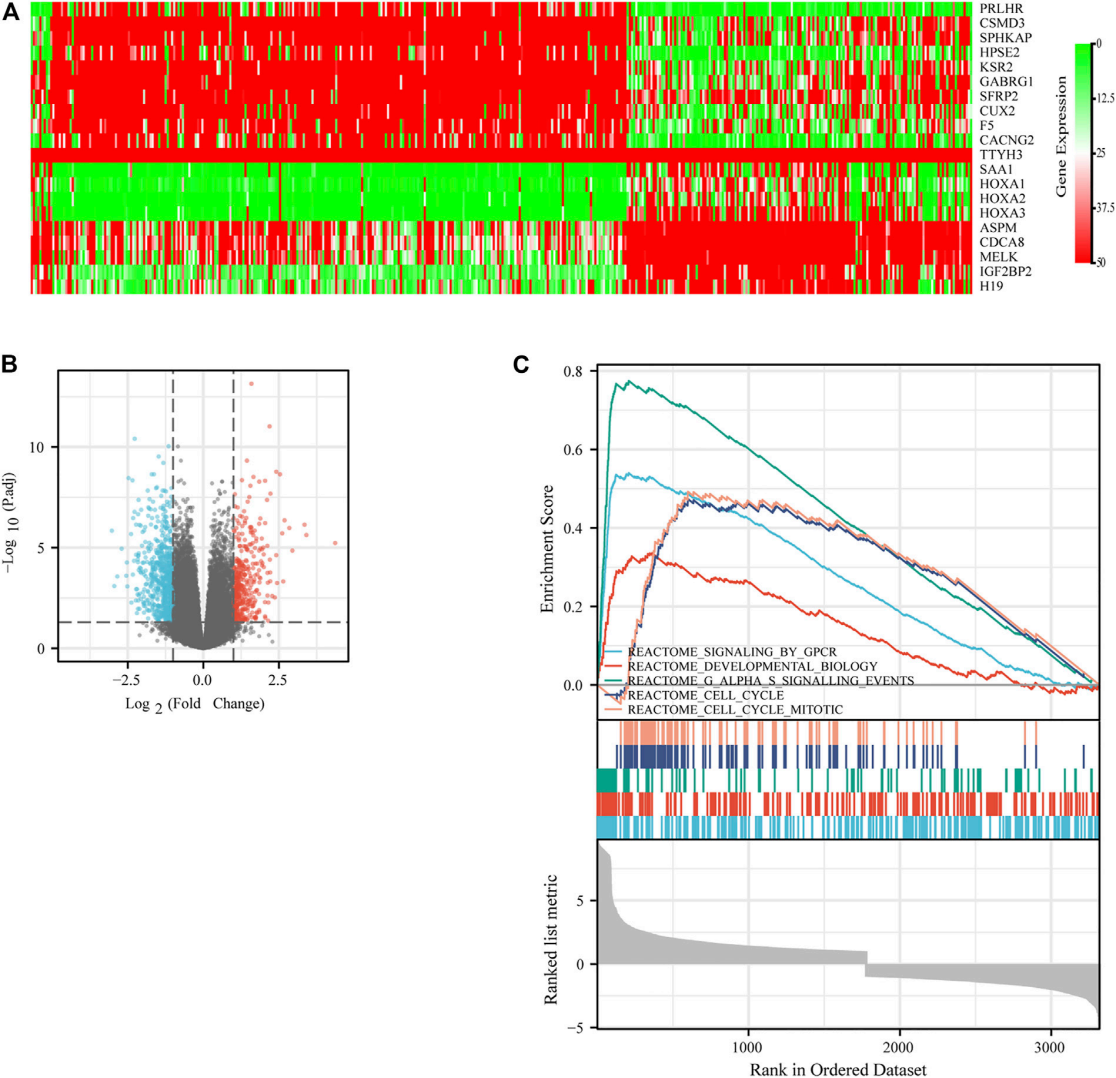
Differentially expressed genes (DEGs) related to IGF2BP3 in gliomas. **(A)** Heatmap of the DEGs. Red scattered dots indicate highly expressed DEGs with the adjusted *p* value < 0.05, and green ones indicated low expressed DEGs with the adjusted *p* value < 0.05. **(B)** Volcano plot of the DEGs. **(C)** Top five significant cellular processes and pathways in GSEA analysis.

Since the expression of IGF2BP3 is significantly associated with patients’ prognosis, we investigated the potential biological process involved in molecular heterogeneity. Patients were divided into low-IGF2BP3 group and high-IGF2BP3 group according to the cutoff value in the survival analysis. The DEGs were analyzed using the Limma R package. Further, log2-fold change (FC) > 2 and adjusted *p* value < 0.05 were used to obtain differential mRNA. A total of 946 differentially expressed genes (DEGs) [log2 (fold change) ≥2 or log2 (fold change) ≤–2, and adjusted *p* < 0.05], including 567 upregulated genes and 379 downregulated genes, were found to be significantly associated with IGF2BP3 expression. The identified DEGs were shown by the Volcano plot (Figure 8B). The top 10 upregulated genes and top 10 downregulated genes were plotted as a heatmap (Figure 8A).

**FIGURE 9:**
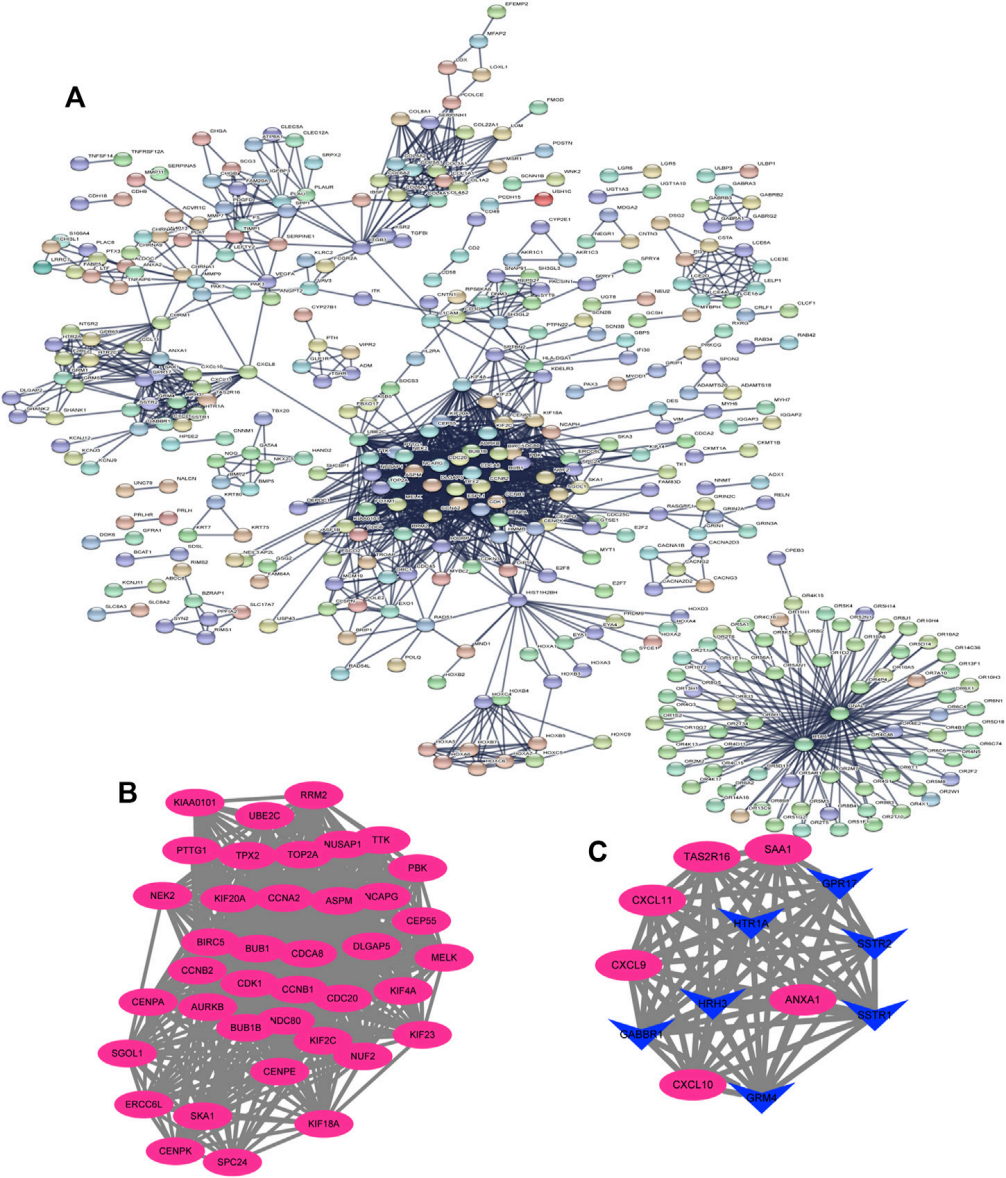
Based on the STRING database and Cytoscape software, protein–protein interaction (PPI) networks of the differentially expressed genes (DEGs) were constructed, followed by modular analyses. **(A)** PPI networks of DEGs generated using the STRING database. **(B, C)** Significant gene module in the PPI networks.

The DEGs were then analyzed using GSEA to determine the signal pathways involved. We found that the DEGs related to the group of high IGF2BP3 expression were mainly enriched in G protein−coupled receptors (GPCR) signaling, cell cycle, G-alpha-S signaling, and cell cycle mitosis (Figure 8C). Subsequently, the PPI analysis was performed on the DEGs, and 335 nodes and 1,627 edges (Figure 9A) were formed. Then, the corresponding gene module was obtained through MCODE in Cytoscape. The score of module 1 was 31.179, with 40 nodes and 608 edges (Figure 9B). The score of module 2 was 14, with 14 nodes and 91 edges (Figure 9C). In addition, we also selected the top 10 hub genes through the cytoHubba App in Cytoscape (Table 2). These results might give us some insights into the cellular biological effects related to IGF2BP3.

**TABLE 2:** Top 10 hub genes.

Gene	MCC score	Type
CDK1	58	up
CDC20	55	up
CCNB1	53	up
BUB1	51	up
CCNB2	50	up
CDCA8	48	up
BUB1B	47	up
KIF2C	46	up
BIRC5	43	up
NDC80	43	up

## Discussion

Glioma is a common primary central nervous system tumor. Approximately 100,000 new patients are diagnosed every year worldwide (Hottinger et al., 2014). The most frequent type of malignant glioma is glioblastoma multiforme (GBM), accounting for 74.6% of all malignant brain tumors and 24.7% of all primary brain tumors (www.agta.org) (Xiao et al., 2020). Despite numerous studies regarding its pathogenesis, the treatment of malignant glioma continues to be challenging, and patient prognoses remain poor. The current treatment for patients with GBM includes surgical resection followed by radiotherapy and adjuvant temozolomide chemotherapy, but fails to provide significant benefits primarily owing to its high proliferative potential, infiltrative growth, and high recurrence rate (Rajesh et al., 2017). Since GBM is highly heterogeneous, a more thorough study of the molecular characteristics of GBM should be performed. Most of the research on gliomas focused on the transcriptional control of gene expression (Mclendon et al., 2008; Verhaak et al., 2010). However, the role of post-transcriptional RNA modifications in cancer progression has just begun to be appreciated.

RNA modifications, known as RNA epigenetics, were present in almost all cellular RNAs. More than 170 different types of post-synthesis modifications have been found in RNAs at present (Boccaletto et al., 2018; Huang H. et al., 2020). However, the mechanisms and functions of RNA modification remain largely unknown. RNA modifications play a critical role in regulating various biological functions, such as RNA processing, transcription, splicing, stability, and translation (Han et al., 2020). RNA modifications are reversible and dynamic, allowing for cellular adaptation to changes in the microenvironment (Huang A. Z. et al., 2020). Of the known RNA modifications, N6-methyladenosine (m^6^A) is one of the most abundant internal messenger RNA modifications found in eukaryotes.

Although m^6^A was discovered around 60 years ago, its function and mechanism were unclear until the first RNA demethylase, fat mass, and obesity-associated protein (FTO) were first detected in 2011 (Jia et al., 2011; Lan et al., 2019). Since then, increased attention has been paid to the function of m^6^A (Desrosiers et al., 1974; Jia et al., 2011; Lan et al., 2019). Accumulating evidence reveals that m^6^A modification is involved in cancer initiation and progression (Li et al., 2017; Zhang et al., 2017; Chen et al., 2018; Liu et al., 2018). Importantly, m^6^A methyltransferases (writers), demethylases (erasers), and m^6^A-binding proteins (readers) are frequently overexpressed in human cancer (Jia et al., 2011) and are strongly associated with tumor prognosis.

Our study found that, except for METTL3, high expression of m^6^A-related genes was associated with poor prognosis. In addition, multivariate Cox regression analysis found that higher expression levels of YTHDC2 and IGF2BP3 were independent negative and positive prognostic factors for OS, respectively. Also, the survival time of patients with high IGF2BP3 expression was significantly reduced.

YTHDC2 and IGF2BP3 are m^6^A readers. The biological importance of m^6^A modification relies on m^6^A-binding proteins (m^6^A readers) because they interpret information related to RNA methylation modification and participate in the translation and degradation of downstream RNA (Wang et al., 2014).

The most well-studied RNA “reader” is the YTH domain-containing family, including YTHDF1, YTHDF2, YTHDF3, YTHDC1, and YTHDC2. (Liao et al., 2018). YTHDF1 is overexpressed in human colon cancer tissues, and its expression is associated with a poor prognosis of colon cancer (Nishizawa et al., 2018). Another study found that YTHDC2 was highly expressed in human colon cancer tissues; a significantly positive correlation was detected between YTHDC2 expression and colon cancer stage, suggesting that YTHDC2 was potentially a diagnostic marker and target gene for treating colon cancer (Tanabe et al., 2016).

In glioma research, a bioinformatics analysis in the CGGA microarray and RNA sequencing databases showed that the levels of YTHDC2, YTHDF1, YTHDF2, and YTHDF3 were elevated in gliomas (Wang et al., 2021). Recently, Xu et al. (Xu et al., 2020) conducted a study on glioma data in TCGA and found that YTHDF1 was associated with glioma progression and high YTHDF1 expression in gliomas was associated with worse OS. The characterization of the YTHDFs as m^6^A readers provided profound insights into our understanding of the effects of m^6^A modification (Huang et al., 2018).

Besides the YTH domain-containing proteins, IGF2BPs including IGF2BP1/2/3 were newly added to the catalog of m^6^A readers (Huang et al., 2018). IGF2BP3 is evolutionarily conserved and can participate in the transport, translation, and conversion of mRNAs by targeting the coding regions of mRNAs (Lederer et al., 2014).

IGF2BP3 is a recognized oncofetal protein present in fetal tissue and not normally expressed in adult tissues. As an RNA-binding protein (RBP), high expression of IGF2BP proteins has been observed in numerous cancer tissues including ovarian clear cell carcinoma, endometrial carcinoma, cervical cancer, lung cancer, renal cell cancers, and glioblastoma (Degrauwe et al., 2016). Many studies identified high IGF2BP3 as an unfavorable prognostic factor for tumors. Research showed that IGF2BP3 expression could be used as a prognostic biomarker for patients with OCCC (Köbel et al., 2009; Liu et al., 2019). In the research on the colon, IGF2BP3 was demonstrated as a predictor of progression and unfavorable prognosis in colon cancer (Lochhead et al., 2012; Yang et al., 2020).

The role of RBPs in glioma progression is only starting to be unveiled. IGF2BP3 was first found to be dramatically upregulated in GBMs, wherein this protein exerted its pro-oncogenic functions through the translational activation of the IGF-2 mRNA (Suvasini et al., 2011). Accumulating evidence indicated that abnormal IGF2BP3 expression had a prognostic value in brain tumors. Zhang obtained mRNA transcription information and clinical data of gliomas from the TCGA database and found that the expression of RAB42, SHOX2, IGFBP2, HIST1H3G, and IGF2BP3 negatively correlated with 5-years OS; also, IGF2BP3 expression significantly positively correlated with glioma grades (Zhang et al., 2019). Alessandro examined IGF2BP3 expression in a series of 135 patients with high-grade gliomas (grades III and IV). They found IGF2BP3-positive high-grade gliomas showed shorter OS and confirmed the role of IGF2BP3 as a marker of poor outcomes (Del Gobbo et al., 2015). Wang analyzed the transcriptome alterations of RBPs and found that the expression levels of SNRPN and IGF2BP3 were significantly associated with the OS of patients in all grades (Wang et al., 2019).

IGF2BP3 was also an epigenetic regulator, which could affect the fates of mRNA in an m^6^A-dependent manner (Bi et al., 2019). Zhou. et al. investigated all m^6^A RNA methylation regulators in colon cancer and found that the high expression of IGF2BP3 was associated with cancer progression and bad prognosis based on TCGA databases. Additionally, they indicated that IGF2BP3 repressed the S phase as well as the proliferation of colon cancer by reading m^6^A modification of CCND1 (Yang et al., 2020).

In our research, the multivariate Cox regression analysis revealed that higher IGF2BP3 expression was associated with poor OS among all m^6^A-associated genes in GBM, and IGF2BP3 expression correlated with the tumor grade. IDH mutation is a major driver of mutations in gliomas; gliomas with IDH mutation have a better prognosis than IDH wild-type gliomas. Our study revealed that IGF2BP3 expression was upregulated in IDH wild-type tumors.

These findings were consistent with previous studies; however, the cellular functions of IGF2BP3 should be elucidated further. The regulation of mRNA fate by IGF2BP3 in gliomas remains less well understood. Whether this phenomenon depends on the activity of IGF2BP3 in m^6^A reading remains unknown.

At present, the research on m^6^A RNA methylation in tumors is still in the early stage. The imbalance of m^6^A levels and modified proteins seems to exert a double-edged sword effect on cancer suppression and promotion. For example, research indicated that m^6^A modification was key to self-renewal and carcinogenesis of glioma stem cells (GSCs). Knockdown of METTL3 or METTL14 promoted the proliferation, self-renewal, and tumorigenesis of GSCs (Cui et al., 2017), indicating that METTL3 was possibly a suppressor gene in GBM. However, Visvanathan obtained the opposite result that METTL3 expression was significantly elevated in GSCs (Visvanathan et al., 2018). The elevated METTL3 expression was associated with the clinical aggressiveness of malignant gliomas (Li et al., 2019). Therefore, the function of m^6^A might be more complex and extensive than reported in existing studies. The results of this study are expected to provide in-depth insights into tumor occurrence and development. They are of great significance in further exploration of the target gene of m^6^A methylation.

Since the expression of IGF2BP3 is significantly associated with patients’ prognosis, we investigate the potential biological process involved in the molecular heterogeneity. In our study, 946 DEGs, including 567 upregulated genes and 379 downregulated genes significantly related to IGF2BP3 expression, were identified. The DEGs were analyzed using GSEA. We found that the DEGs related to the group of high IGF2BP3 expression were mainly enriched in GPCR signaling, cell cycle, G-alpha-S signaling, and cell cycle mitosis. The top 10 upregulated genes and the top 10 downregulated genes were plotted as a heatmap, which provided some promising directions for future investigation.

Recently, m^6^A methylation−related genes have shown great potential in the prognostic prediction of cancer. In the present study, we comprehensively analyzed the expression of m^6^A-regulated genes in gliomas and their association with clinical characteristics. Notably, we found that IGF2BP3 expression predicted a poor prognosis and was an independent risk factor for OS of patients with glioma. Our study highlighted IGF2BP3 as a potential prognostic predictor as well as a therapeutic target for glioma. We analyzed the DEGs related to IGF2BP3 and tumor-related pathways regulated by differentially expressed m^6^A-modified genes. More studies are needed for further clarification of these findings.

## Data Availability

The original contributions presented in the study are publicly available. This data can be found here: http://xena.ucsc.edu/ and http://www.cbioportal.org. For detailed methods, please refer: https://www.biorxiv.org/search/326470v6, and https://pubmed.ncbi.nlm.nih.gov/23550210/.
